# Prevalence of 25-Hydroxyvitamin D (Vitamin D) Deficiency in a Group of Infertile Women from Baghdad City

**DOI:** 10.1155/2023/6597730

**Published:** 2023-06-14

**Authors:** Muthana Anad Majid, Wafaa Nasser Hassan, Amna Fadhil Ridha

**Affiliations:** ^1^Department of Basic Sciences, College of Dentistry, Mustansiryiah University, Palestine Street, Baghdad, Iraq; ^2^Department of Basic Sciences, College of Medicine, Ibn Sina University for Medical and Pharmaceutical Sciences, Qadisya District, Baghdad, Iraq; ^3^Department of Obstetrics and Gynecology, College of Medicine, Mustansiryah University, Yarmouk Hospital, Baghdad, Iraq

## Abstract

**Background:**

Infertility is a common issue affecting a large number of Iraqi women of reproductive age. The relationship between vitamin D deficiency and infertility has previously drawn the attention of gynecologists, and an increasing number of vitamin D testing has been requested.

**Methods:**

120 women were enrolled in this study between April 2019 and April 2020. Patients were divided into two groups comprising sixty women complaining of infertility, with the other 60 women being fertile and enrolled as controls. All patients were assessed for vitamin D level.

**Results:**

In the fertile study group, patients with deficient, insufficient, and sufficient level of vitamin were 28%, 23%, and 48%, respectively (these numbers were rounded to the nearest whole digit, as the numbers for the infertile group were given with that level of precision), whereas the infertile study group showed a statistically significant (*p* value = 0.002) distribution of vitamin levels with 50%, 35%, and 15% of women being deficient, insufficient, and sufficient, respectively.

**Conclusions:**

Vitamin D is significantly deficient in infertile patients which suggests a possible, positive impact if vitamin D is considered in the management of female infertility. Further study with more participants is highly recommended.

## 1. Introduction

Numerous human disorders and diseases are associated with vitamin D deficiencies, including diabetes, autoimmune diseases, cardiovascular diseases, and cancer [1–3]. However, despite inconclusive data [4], vitamin D has also been shown to be of great importance in conditions associated with infertility [5, 6], making vitamin D deficiency a global pandemic [7]. In Iraq, infertility is an issue affecting many married couples and nowadays its relation with vitamin D deficiency is drawing the attention of Iraqi physicians [8].

Vitamin D receptors are expressed in many tissues that respond to vitamin D including parathyroid glands, skeletal tissue, bones, kidneys, and intestines [2, 3, 9, 10]. The biological action of the sites that express these receptors is affected by vitamin D levels. Reproductive tissues are also enriched with vitamin D receptors (VDRs) that control the biological action of vitamin D. In fact, the ovaries have been shown to express vitamin D receptor-messenger RNA (VDR mRNA) [11]. The vitamin D receptors are also found in the endometrium [11, 12], and researchers found that the endometrium is capable of extra-renal synthesis of vitamin D in its active form [13] indicating the importance of this vitamin in the reproductive tissue. These observations all suggest that vitamin D plays an important role in steroidogenesis of sex hormones [10, 14].

It is thought that in humans, nearly 80–90% of vitamin D is synthesized upon exposure to sunlight, with the other sources of vitamin D being diet and supplements (10–20%) [5]. The precursor of the steroid hormone vitamin D is an intermediate of the cholesterol pathways, 7-dehydrocholesterol. Prior to its conversion to cholecalciferol (vitamin D), 7-dehydrocholesterol is metabolized in the liver and converted into pro-vitamin D via the enzyme 25-hydroxylase (25(OH)D) [15] where it is released into circulation bound to vitamin D binding protein (VDBP). Therefore, the concentration of this enzyme can be used in the laboratory to analyze the level of patient's vitamin D levels.

It has been found that the enzyme 1,25(OH)2 D_3_ also contributes to the production of progesterone and estrone [16]. This enzyme has also been found to control the secretion and expression of human chorionic gonadotrophins, as well as increasing placental sex steroid production [13, 16]. Studies have also demonstrated that the active form of vitamin D, calcitriol, which is synthesized in the kidneys, is crucial in the development of the uterus and endometrium via regulating the expression of *HOXA10* gene in endometrial stroma cells. *HOXA10* is critical for the implantation of fertilized ovum into the endometrium [16, 17].

In this study, we assessed the prevalence of vitamin D deficiency in Iraqi women with primary and secondary infertility during their reproductive age.

## 2. Materials and Methods

### 2.1. Patient Cohort

60 women with unexplained infertility who attended our laboratory and private clinic in Baghdad (Iraq) between April 2019 and April 2020 were enrolled in this study along with 60 healthy women as controls. This study only included infertile women between the ages of fifteen and forty-three who were unable to conceive after at least a year of regular, unprotected sexual activity, despite normal ovarian reserves and typical tubal patency, and whose partners' sperm analysis findings were normal.

Smoking, endometriosis, ovarian lumps, hyperprolactinemia, hypogonadotropic hypogonadism, menopause, congenital adrenal hyperplasia, androgen-secreting tumors, and Cushing's syndrome cases were all excluded from the study. Individuals with systemic diseases like thyroid and kidney problems, as well as high blood pressure, were also disqualifiers. Control women were selected from healthy patients with past history of child birth. None of the patients have been supplemented with vitamin D prior to tests.

Parameters such as physical examination and their weight, height, and waist circumference (WC) measurements were taken for all subjects.

Patients were divided into two groups, 60 infertile women and 60 fertile controls. Both groups were similar in age ranging from 15 to 43 years, with majority at the range of 20–26 years. All patients were screened for vitamin D levels.

### 2.2. Vitamin D Measurements

Three milliliters of venous blood were collected, and a quantitative determination of total 25(OH)D/D levels in the patients' sera was conducted. Tests were run immediately using fluorescence immune assay (FIA) on i-CHROMA™ AFIAS auto-analyzer [18, 19]. No special conditions, such as fasting, were needed before phlebotomy.

### 2.3. Analysis and Statistics

Endocrine Society Clinical Practice Guidelines [20] define vitamin D deficiency as a 25(OH) vitamin D level of <20 ng/mL, vitamin D insufficiency as a 25(OH) vitamin D level of 21–29 ng/mL, and vitamin D sufficiency as a 25(OH) vitamin D more than 30 ng/mL.

However, other reference ranges of vitamin D were used slightly different of the above as follows: deficient—>20 ng/mL; insufficient—20–29 ng/mL; and sufficient—≥30 ng/mL [21]. Within the normal range, two groupings were defined: low normal (lower limits of normal range) was between 30 and 35 ng/mL, whereas fully normal (higher limits of normal range) was above 35 ng/mL. Statistical distributions were calculated in GraphPad Prism using contingency statistical analysis.

## 3. Results

A total of 120 women were assessed for their vitamin D levels, categorized into two groups with 60 patients who were infertile and 60 fertile women as a control group. The percent of infertile patients that had deficient, insufficient, and sufficient level of vitamin D was 50%, 35%, and 15%, respectively (Table 1). In the control group, however, the percent of women that had deficient, insufficient, and sufficient level of vitamin D level was 23%, 28%, and 48%, respectively. When the sufficient vitamin D category was subdivided into low normal and fully normal, 44 of the infertile women in the normal range were in the low normal group, whereas only 21% of the fertile women had low normal vitamin D.

Contingency statistical analysis (Using GraphPad Prism) showed that the distribution of vitamin D levels in infertile women was significantly different from that in the fertile control group (*p*=0.0014).

## 4. Discussion

Infertility is an ongoing debate in public health, and it strikes more than 48 million couples around the globe [15]. It has serious psychological, medical, and financial effects. In a case-cohort research done at a teaching hospital in Pittsburgh, Pennsylvania, researchers looked at whether the amounts of vitamin D in women's samples who visited this hospital are linked to the number of live births. It was discovered that cases with vitamin D readings over 30 ng/mL had a higher chance of live births than those with lower levels [22]. In Iraq and the Middle East in general, the exposure to sunlight is very low despite the all year round sunlight availability. This is due to several causes including the cultural and religious aspects that most women cover themselves [23]. However, regardless of this reason, most Iraqis try to avoid the sunlight all the time due to the hot environment in this region (reaching more than 50°C during summer keeping in mind that summer is a very long season here [24]). This justifies the need for more studies about the implication of vitamin D deficiency on conception in Iraq.

Normal vitamin D levels tend to have a positive effect on fertility, and they may also increase the likelihood of having a healthy gestation. Extensive body of research has linked vitamin D deficiency to an increased risk of premature delivery, gestational diabetes, pre-eclampsia (very high blood pressure during pregnancy), and bacterial vaginosis [25–28].

Endocrine Society Clinical Practice Guidelines defined vitamin D deficiency as a 25(OH) vitamin D level of <20 ng/mL, vitamin D insufficiency as a 25(OH) level of 21–29 ng/mL, and vitamin D sufficiency as a 25(OH) vitamin D level more than 30 ng/mL.

Vitamin D is commonly named as the “sunshine vitamin.” It is generated in the skin in reaction to exposed to sunlight, and it can also be absorbed via food and supplements. Most individuals can produce sufficient vitamin D if they are exposed to sunlight for long enough time. Nevertheless, many females do not receive sufficient sunlight to maintain adequate vitamin D levels. With a handful of naturally vitamin D-rich foods, such as oily fish, milk, and egg yolks [29], it is also challenging to obtain enough dietary vitamin D.

Moreover, there are other factors that influence vitamin D levels as well, including obesity and dark complexion. Vitamin D deficiency is common among women attempting to conceive for these and other reasons.

A number of studies highlight the importance of vitamin D in conceiving, placentation, the course of pregnancy, and the well-being of the progeny. Low levels of vitamin D are common among women of reproductive age [30].

Hypovitaminosis D is associated with infertility and a number of adverse pregnancy outcomes [5, 31]. In addition, pre-pregnancy vitamin D values above 75 nmol/L (30,05 ng/mL) have been linked to a higher probability of pregnancy, a decrease in loss of pregnancy, and a rise in the number of live births [32].

The importance of vitamin D production at the feto-maternal interface is emphasized by the expression of *α*-hydroxylase in the decidua and placenta [33–35]. Overall, vitamin D promotes placental growth and action by modulating placental calcium transport and by exhibiting immunomodulatory activities, which are essential for maintaining pregnancy [36].

Early in pregnancy, the quick onset of VDR and *α*-hydroxylase CYP27B1 in the decidua and placenta plays an essential mission for vitamin D in formation, involving implantation and the growth of the placenta [37–39]. Vitamin D binding to VDR has been shown to adjust important target genes, such as *HOXA10*, which are essential for endometrial development, uterine receptivity, and implantation [40].

Additionally, as a direct action in decidualization and placentation, vitamin D could indirectly affect growing and placentation through its immunomodulatory effects. Many years ago, it was hypothesized that the immunosuppressive effects of vitamin D are important for healthy pregnancy starting from implantation until delivery which might avoid a maternal immune reaction toward the embryo carrying paternal DNA [38].

During pregnancy, decidual production of vitamin D is likely working on restraining NK cells, DCs, macrophages, and T-cells leading to immune tolerance [41, 42]. It is well known that vitamin D reduces effect of Th1 cytokines while promoting Th2 cytokines, and thus it may indulge the process of implantation [42, 43].

Though data regarding vitamin D and fertility are inconclusive, a good number of research studies have shown that vitamin D readings in the blood of 30 ng/mL or higher have been linked to increased pregnancy rates [18, 44–46]. Among populations of predominantly Caucasian and non-Hispanic white females, individuals with an adequate vitamin D level had been four times more probable to become pregnant via IVF compared to those with an insufficient vitamin D level, according to two articles [18, 45]. Rudick et al. found that donor egg patients with typical vitamin D values had increased pregnancy incidents in comparison to those with low vitamin D values [47]. Therefore, continuing to take a vitamin D supplementation during antenatal period is beneficial for both mother and fetus. Research demonstrates that taking 2,000 to 4,000 IU of vitamin D is secure and not harmful for pregnant women to achieve normal vitamin D levels to prevent vitamin D deficiency in neonates [48, 49].

Recent research [50] showed that vitamin D supplements stimulated the survival and development of antral follicles and the maturation of oocytes. It is well noted that there are correlations among fertility, seasonal variations, and geographical areas. Nowadays, it is evident that these variations are owing to UV-dependent fluctuations in vitamin D levels [51, 52].

Injection of vitamin D increases uterine heaviness and promotes decidualization of the endometrium in pseudo-pregnant mice, which may suggest that vitamin D is involved in a crucial step in blastocyst implant [53, 54]. This study demonstrates that vitamin D insufficiency is a risk factor and latent cause of infertility among Iraqi women.

This study employed the use of a sensitive auto-analyzer with population-based, non-interventional, and prospective design. Furthermore, the target patients are Iraqi women who are seeking pregnancy with no obvious reasons for infertility.

A recent study on a sample of women from Hilla city showed that vitamin D is deficient in 76% of enrolled women in their reproductive age. The study pointed out a possible link of vitamin D deficiency with infertility [8].

One study recruited all vitamin D deficient infertile Saudi women presenting to the infertility department, King Fahad Medical Center, and 82.1% of vitamin D deficient women experienced IVF as opposed to 36.4% of vitamin D insufficient women having a meaningful difference of *p* value = 0.001 [53].

In a 2013 study by Garabedian et al., 54.9% of participants had inadequate 25(OH)D levels, while 45.1% had normal readings [46]. Women having adequate levels had substantially larger probability of clinical pregnancy per IVF cycle initiated (52.5% vs. 34.7%; *p* < 0.001) than those with inadequate levels (34.7%). Implantation rates seem to be greater in the group with adequate levels of 25(OH)D; however, the difference was statistically insignificant. Recently, a study hypothesized that vitamin D deficiency may negatively impact pregnancy rates via the endometrium, as vitamin D deficiency was not associated with ovarian influence features or markers of embryo quality. Vitamin D may influence IVF cycle outcomes through the endometrium VDR, which is found in the endometrium and has a crucial role in triggering the innate immune response [55].

Vitamin D deficit as well as inadequacy levels had negatively impacted the success of *in vitro* fertilization (IVF), leading to decreased pregnancy rates among Arabian females in reproductive age. Vitamin D supplements may improve treatment outcomes for infertile individuals undergoing IVF and thus were advised for infertile women [6].

Vitamin D deficiency was identified in 53.2% (266/500) of participants in one study [16], while vitamin D insufficiency was reported in 30.8% (154/500) of participants. Only 16% (80/500) of women had adequate vitamin D, and vitamin D deficiency and insufficiency are widespread among females using reproductive technology. In addition, serum vitamin D levels correlate with IVF results.

Many of the subsequent studies [55, 56] found a positive relation between vitamin D levels and IVF success. Recent evidence suggests that vitamin D deficiency (20 ng/mL) affects pregnancy success in females attempting single embryo transfer (SET) on day 5 (blastocyst stage). Lower clinical pregnancy rates were attributed to vitamin D deficiency's detrimental impact on endometrial receptivity [57].

Our study indicates that vitamin D deficiency and insufficiency are highly dominant where 50% of infertile women had vitamin D deficiency, 35% were insufficient, and only 15% had sufficient level of vitamin D with a *p* value of 0.0014. However, off those 15%, sufficient level, there were approximately 44% at lowest sufficient. This gives a good idea about the importance of this vitamin for women trying to conceive. Although there are many studies in Iraq linking vitamin D deficiency with infertility, to our knowledge, this is the first study, in Baghdad, that links vitamin D deficiency directly with infertility.

## 5. Conclusion

These data demonstrate a relationship between vitamin D and infertility in the sample size we used in this article. However, a larger sample of patients and more comprehensive research are definitely recommended for future studies. There certainly is a need for further investigation of the lowest level of vitamin D that is acceptable for fertility (now it is 30 ng/mL).

Women pursuing pregnancy should be advised to take vitamin D supplementation when found deficient or insufficient, and this is relatively safe and inexpensive. A further follow-up study of the patients after supplementation with vitamin D and fixing the levels to sufficient amount is to be conducted to evaluate these findings.

## Figures and Tables

**Table 1:** Vitamin D levels in infertile Iraqi women compared to healthy women.

	Insufficient (<20 ng/mL)	Deficient (20–29 ng/mL)	Sufficient (>30 ng/mL)	Low normal^∗^ (30–35 ng/mL)
Patients	30 (50%)	21 (35%)	9 (15%)	4 (44%)
Control	14 (23%)	17 (28%)	29 (48%)	6 (21%)

^*∗*^Number of women with low normal vitamin D levels (between 30 and 35 ng/mL) with the percentage calculated against the number of women with normal vitamin D, control, rather than against the whole cohort.

## Data Availability

The datasets used and/or analyzed during the current study are available from the corresponding author on reasonable request.
